# Physicochemical, rheological, sensory, microbiological, and oxidative properties of canned pâtés reformulated with hydrated pea protein as a fat replacer

**DOI:** 10.1002/jsfa.70331

**Published:** 2025-11-17

**Authors:** Pamela Cristiele Oliveira Trindade, Bibiana Alves dos Santos, Géssica Hollweg, Milena Padilha, Priscila Rossato Fracari, Sarita Correa Rosa, Alexandre José Cichoski, José Manuel Lorenzo, Paulo Ricardo de Matos, Márcio Vargas‐Ramella, Paulo Cezar Bastianello Campagnol

**Affiliations:** ^1^ Department of Food Science and Technology Universidade Federal de Santa Maria Santa Maria Brazil; ^2^ Centro Tecnológico de la Carne de Galicia Ourense Spain; ^3^ Area de Tecnología de los Alimentos, Facultad de Ciencias de Ourense Universidade de Vigo Ourense Spain; ^4^ Department of Civil Engineering Universidade do Estado de Santa Catarina (UDESC) Joinville Brazil; ^5^ Centro de Educação Superior da Região Sul—CERES Universidade do Estado de Santa Catarina (UDESC) Laguna Brazil

**Keywords:** pea protein, low‐fat meat products, lipid oxidation, shelf life, sensory quality, healthier meat products

## Abstract

**BACKGROUND:**

The objective of this study was to evaluate the technological and sensory feasibility of partially replacing pork fat with hydrated pea protein in canned pâtés, using different hydration levels. Six formulations were prepared: one Control (100% pork fat) and five treatments (G_1:1_, G_1:2_, G_1:3_, G_1:4_, and G_1:5_), in which 50% of the fat was replaced with hydrated pea protein at increasing protein‐to‐water ratios of 1:1, 1:2, 1:3, 1:4, and 1:5, respectively.

**RESULTS:**

The reformulation led to an approximate 50% reduction in fat content and up to a 24% increase in protein content (*P* < 0.05). Formulations G_1:3_, G_1:4_, and G_1:5_ exhibited satisfactory technological performance. Additionally, these samples exhibited favorable sensory attributes, including a soft texture, good spreadability, a pleasant flavor, a homogeneous appearance, and a pink color, resulting in acceptance levels comparable to those of the Control (*P* > 0.05). Rheological analyses revealed lower flow resistance and reduced elastic modulus in these formulations (*P* < 0.05), contributing to improved spreadability. All samples maintained microbiological stability throughout 60 days of refrigerated storage (*P* > 0.05). Although the reformulated products showed increased thiobarbituric acid reactive substances (TBARS) values (*P* < 0.05), the levels remained low and did not compromise the sensory quality or safety of the product.

**CONCLUSION:**

The results indicate that partially replacing pork fat with hydrated pea protein concentrate is a viable strategy for developing healthier pâtés, while preserving the product's technological, sensory, and microbiological quality. © 2025 The Author(s). *Journal of the Science of Food and Agriculture* published by John Wiley & Sons Ltd on behalf of Society of Chemical Industry.

## INTRODUCTION

Canned meat products, such as pâtés, are valued for their convenience, microbiological stability, long shelf life, and sensory appeal – particularly their creamy texture, spreadability, and pleasant flavor. They are also widely used as nutritional supplements due to their high protein content.^1^, ^2^ However, these products typically contain high levels of animal fat, rich in saturated fatty acids.^3^, ^4^ Excessive intake of these fats is linked to cardiovascular disease, obesity, and metabolic syndromes, posing a challenge for the meat industry.^5^

To address this, various fat‐reduction strategies have been investigated, especially those involving the partial or total replacement of animal fat with plant‐based ingredients.^6^ Among them, pea protein has gained increasing attention due to its favorable technological and nutritional properties. It offers excellent emulsifying capacity, water retention, and protein network formation – attributes essential for preserving product texture and stability.^7^ Additionally, its balanced profile of essential amino acids, particularly lysine, leucine, and phenylalanine, enhances the nutritional quality of the final product.^8^ More recently, studies have explored the synergistic use of pea protein with polysaccharides to produce solid‐fat mimetics with similar properties to animal fat,^9^, ^10^ reinforcing its multifunctional potential.

The hydration process of plant proteins is known to influence their structural conformation, functional performance, and sensory behavior. Hydration promotes partial unfolding of protein molecules, improving solubility, emulsifying activity, and network‐forming ability – key factors in fat replacement applications.^7^, ^11^ However, hydration levels must be optimized, as excessive water addition can impair texture and flavor, while insufficient hydration may result in a gritty mouthfeel and reduced protein functionality.

Previous studies have demonstrated the successful application of pea protein in products such as burgers, sausages, and breaded meats, yielding improvements in nutritional, sensory, and functional characteristics.^10^, ^12^, ^13^ However, its targeted use as a hydrated fat replacer in canned meat products remains largely underexplored. In particular, there is a lack of data on how different hydration ratios of pea protein affect the technological, rheological, sensory, oxidative, and microbiological properties of such products during storage.

In this context, the objective of this study was to evaluate the technological and sensory feasibility of partially replacing pork fat with hydrated pea protein concentrate in canned pâtés, examining the influence of different protein‐to‐water ratios. The impacts of these hydration levels on essential quality attributes – such as technological performance, texture, nutritional profile, microbiological stability, lipid oxidation, and overall sensory acceptance – were assessed. To our knowledge, this is the first study to compare multiple hydration ratios of pea protein in a heat‐treated, shelf‐stable meat matrix, offering novel insights into formulation strategies for healthier pâtés. Consequently, these findings support the development of healthier meat products aligned with contemporary consumer preferences and market trends.

## MATERIALS AND METHODS

### Raw materials

Pork meat (semimembranosus muscle from fresh post‐mortem pork ham; post‐mortem period ~24 h; pH 5.6) with moisture: 76.08 ± 1.2%, lipids: 4.43 ± 0.4%, protein: 18.01 ± 0.9%, and pork fat (moisture: 17.28 ± 0.5%, lipids: 75.39 ± 2.1%, protein: 6.54 ± 0.1%) were purchased from a local market. The muscle was trimmed of visible connective tissue and subcutaneous fat before use. Pea protein concentrate (moisture: 2.5%, carbohydrates: 36.7%, protein: 60%, ash: 0.8%, pH 6.16, color: L* = 91.59; a* = 1.00; b* = 17.79) was donated by Nutrassim (Extrema, MG, Brazil), and the chemical additives and seasonings were donated by Ad Foods (Imbituba, SC, Brazil).

### Preparation of canned pâté

The Control pâté was formulated with pork meat (65%), pork fat (20%), sodium chloride (2%), carrageenan (0.5%), California seasoning (0.5%; composed of salt 91.6%, sugar 5.1%, monosodium glutamate 0.8%, garlic powder 1.2%, onion powder 0.8%, clove oil resin 0.3%, and cinnamon essential oil 0.2%), sodium nitrite (0.015%), sodium tripolyphosphate (0.5%), sodium erythorbate (0.025%), and water (11.46%). In the reformulated treatments, 50% of the pork fat in the Control was replaced with hydrated pea protein concentrate at different protein‐to‐water ratios: 1:1 (G_1:1_), 1:2 (G_1:2_), 1:3 (G_1:3_), 1:4 (G_1:4_), and 1:5 (G_1:5_). Hydration was performed by dispersing the pea protein concentrate in water preheated to 50 °C, followed by homogenization of the suspension using a Turrax homogenizer Ultra‐Turrax MA 102 (220 VAC, 650 W, max ~27 000 rpm; Marconi, Piracicaba, SP, Brazil) at ~15 000 rpm for 1.5 min, followed by a 5 min rest at room temperature before incorporation into the formulation.

To prepare the pâté, pork meat and fat were cut into ~5 cm cubes and vacuum‐packed separately. The pork meat was pre‐cooked in water at 80 °C for 30 min, and the fat was pre‐cooked for 10 min. Homogenization was carried out in a cutter (R.I 60, Cutter Industrial Ltd, Chapecó, SC, Brazil), with knife rotation at approximately 1700 rpm and bowl rotation at 22 rpm. Initially, meat and its cooking liquid were mixed for 2 min. Subsequently, pork fat and hydrated pea protein were added, and water preheated to 65 °C was gradually incorporated. The mixture was then homogenized for approximately 3 min, resulting in a uniform mass. The resulting mixture was filled into metal cans (3.00 cm height × 7.50 cm diameter) containing 100 g of product. The cans were sealed using a can sealer (Mocmaq, São Paulo, Brazil), then cooked in a water bath at 80 °C until the internal core temperature reached 72 °C (approximately 30 min), followed by cooling in an ice bath for 20 min before storage at 4 °C. Internal temperature validation was performed in three test cans using thermocouples placed at their geometric center. These cans were excluded from subsequent analyses.

### Analyses performed immediately after processing

#### Chemical composition

Chemical composition was analyzed in triplicate. Lipid content was determined using the chloroform–methanol–water extraction method of Bligh and Dyer,^14^ involving a one‐phase mixture followed by phase separation to isolate the lipid‐containing chloroform layer, which was evaporated to constant weight. Moisture, ash, and protein contents were determined following the Association of Official Analytical Chemists (AOAC) methods 950.46, 920.153, and 992.15, respectively.^15^

#### The pH and water activity (Aw)

For pH determination, 5 g of sample was homogenized with 50 mL of distilled water. Measurements were performed using a pH meter (Model 130 MA; Mettler Toledo, Barueri, SP, Brazil), calibrated with pH 4.0 and 7.0 buffer solutions (Merck, Darmstadt, Germany). Water activity (Aw) was measured with an AquaLab Series 4 TEV (Decagon Devices, Inc., Pullman, WA, USA). Both analyses were conducted in triplicate.

#### Instrumental color

Instrumental color (L*: lightness, a*: redness, b*: yellowness) was measured 15 min after opening the cans. Two cans were evaluated per treatment, with six independent readings taken per can. A CR‐400 colorimeter (Konica Minolta Sensing Inc., Osaka, Japan) was used in spectral reflectance mode with a 10° observer angle, D65 illuminant, and a 1.5 cm diameter aperture.

#### Penetration test

Penetration test was performed using a Stable Micro Systems Texture Analyzer TA‐XT2 (Stable Micro Systems, Godalming, UK), equipped with a 6 mm cylindrical probe (P/6). Test parameters included: pre‐test speed = 1.50 mm s^−1^, test speed = 1.50 mm s^−1^, post‐test speed = 10.00 mm s^−1^, initial probe distance = 8.00 mm, and trigger force = 10.0 × *g*. The probe penetrated the sample to a depth of 15 mm at a constant speed. Hardness, the peak force required for penetration (in newtons), was derived from the force‐time curves. Two cans per treatment were analyzed at room temperature, with five replicates per can. Pâtés were randomly selected and measured directly in the can in a horizontal position.

#### Sensory analysis

The study protocol was approved by the Ethics Committee of the Federal University of Santa Maria (CAAE: 77177324.5.0000.5346), and all participants signed informed consent forms. All pâté samples met the microbiological standards required by Brazilian legislation.^16^ Sensory evaluations were conducted in individual booths illuminated with fluorescent lights (~350 lx). Approximately 15 g of each coded sample (random three‐digit numbers) was served at ~10 °C, in monadic sequence, using a Latin square design.^17^ Panelists were instructed to visually inspect and smell each sample before spreading it on saltine crackers. Between samples, panelists cleansed their palates with room‐temperature water.

##### Acceptance and CATA tests

One hundred regular pâté consumers (aged 18–45 years; 41 males and 59 females) participated in the acceptance and Check‐All‐That‐Apply (CATA) tests. First, consumers rated color, aroma, flavor, texture, and overall liking using a 9 cm unstructured hedonic scale anchored with ‘disliked very much’ on the left and ‘liked very much’ on the right. Second, they completed the CATA questionnaire by selecting descriptors that best characterized the samples. The CATA descriptors included: homogeneous appearance, oily, pink color, unpleasant color, pleasant color, yellowish color, pale color, pea aroma, mild aroma, rancid aroma, vegetal aroma, cooked meat aroma, unpleasant aroma, salty taste, mild taste, pleasant taste, unpleasant taste, pork flavor, pea flavor, rancid flavor, soft, good spreadability, grainy, pleasant texture, poor spreadability, and unpleasant texture.

##### Sensory profile

The sensory profile was evaluated by 15 trained panelists (aged 22–35 years; nine females and six males) following the method described by Pinton *et al*.^18^ Training consisted of two 2 h sessions in which panelists were familiarized with descriptor terms and corresponding references (Supporting Information, Table S1). After training, the panelists rated the samples using a 9 cm unstructured scale anchored with ‘low’ or ‘none’ on the left and ‘high’ on the right. Evaluated descriptors included: homogeneous appearance, pink color, spreadability, vegetal aroma, characteristic aroma, rancid aroma, salty taste, rancid taste, characteristic taste, and vegetal taste.

#### Rheological characterization

Rheological properties were assessed at 5 and 25 °C using a Viscotester iQ Air (Haake, Thermo Scientific, Waltham, MA, USA) equipped with a four‐blade vane spindle (*⌀* = 22.0 mm; height = 11.0 mm). Stress *versus* strain curves were recorded at a constant shear rate of 0.1 s^−1^ for 60 s. Yield stress was defined as the peak stress value, and the corresponding strain was recorded as yield strain. The elastic modulus was calculated as the slope of the linear region of the stress–strain curve, up to one‐third of the yield strain, excluding the initial data point. Two samples were analyzed, and results were expressed as mean ± standard deviation. All measurements were conducted directly in the cans to prevent disruption from sample handling.

### Analyses during storage

#### Microbiological quality

Counts of mesophilic aerobic microorganisms were performed in triplicate on days 1, 15, 30, 45, and 60 of storage, according to ISO 7218.^19^ For each time point, 25 g of sample was collected immediately after the can opening and diluted in 225 mL of 0.1% peptone water (Merck). Serial dilutions were prepared and inoculated by depth plating in sterile Petri dishes using plate count agar (PCA; Merck). After medium solidification, plates were incubated at 37 °C for 48 h. Results were expressed as log colony forming unit (CFU) per gram.

#### Oxidative stability

Thiobarbituric acid reactive substances (TBARS) were measured in triplicate on days 1, 15, 30, 45, and 60 of storage, following the method described by Bruna *et al*.^20^ Results were expressed as milligrams of malondialdehyde (MDA) per kilogram of sample.

### Statistical analysis

All experiments were performed in triplicate (*n* = 3). Physicochemical data were analyzed using a generalized linear model, considering treatments as fixed effects and replicates as a random effect. For analyses during storage, storage time was also considered a fixed effect, and interactions between fixed effects were assessed. Tukey's test was used for mean comparisons with a significance level of 5%.

A mixed linear model was used to analyze sensory acceptance data, with treatments as fixed effects and panelists as random effects. Tukey's test was applied for pairwise comparisons at a 5% significance level. CATA descriptor data were analyzed using correspondence analysis. Principal coordinate analysis was used to evaluate correlations between CATA descriptors and overall liking scores, applying tetrachoric and biserial correlations. Generalized procrustes analysis (GPA) was used to analyze sensory profile data. All statistical analyses were performed using XLSTAT 2019.1 (Addinsoft, Paris, France).

## RESULTS AND DISCUSSION

### Analyses performed immediately after processing

#### Chemical composition, pH, and water activity (Aw)

**Figure 1:**
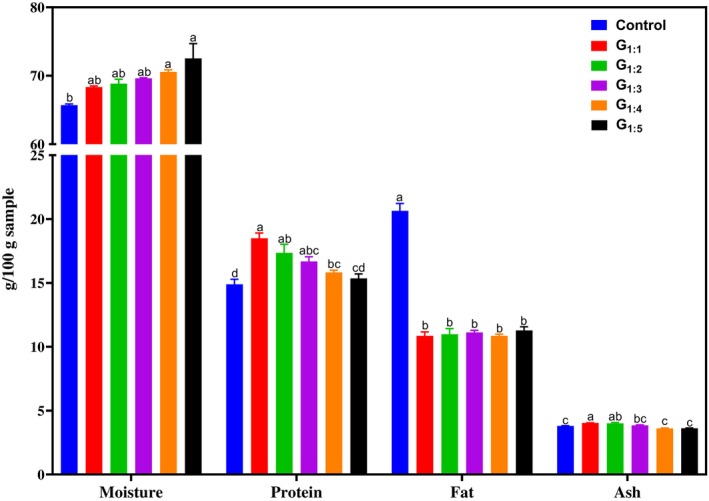
Chemical composition (%) of canned pâtés reformulated with hydrated pea protein at different hydration ratios. Different letters indicate significant differences based on Tukey's test (*P* < 0.05). Error bars depict the standard error of the average. Batches: Control: 100% pork fat; Reformulated pâtés: substitution of 50% pork fat with hydrated pea protein using different protein‐to‐water ratios: 1:1 (G_1:1_), 1:2 (G_1:2_), 1:3 (G_1:3_), 1:4 (G_1:4_), and 1:5 (G_1:5_).

The chemical composition of the pâtés prepared with 50% replacement of pork fat by hydrated pea protein concentrate at different hydration ratios is presented in Fig. 1. Moisture content increased with higher hydration levels of the pea protein concentrate. However, only the G_1:4_ and G_1:5_ treatments exhibited significantly higher moisture levels (*P* < 0.05) than the Control. Protein content significantly increased in treatments G_1:1_ through G_1:4_, ranging from 6.3% (G_1:4_) to 24.3% (G_1:1_) compared to the Control (*P* < 0.05). These findings align with those reported by Lima Guterres *et al*.,^12^ who demonstrated that replacing animal fat with hydrogelled emulsions containing pea protein increased protein levels. This increase contributes to the product's nutritional quality, considering the high biological value of pea protein due to its balanced essential amino acid profile.^7^, ^11^ As expected, the modified treatments exhibited an approximate 50% reduction in fat content relative to the Control (*P* < 0.05), a nutritional improvement given that reducing animal fat intake is an effective strategy for lowering the risk of cardiovascular disease and obesity.^11^ No significant differences (*P* > 0.05) in fat content were observed among the reformulated treatments (G_1:1_ to G_1:5_), indicating that the varying hydration ratios of the pea protein concentrate did not affect the final fat levels in the products. Regarding ash content, only treatments G_1:1_ and G_1:2_ showed significantly higher values than the Control (*P* < 0.05), likely due to the greater proportion of pea protein in these formulations compared to the other modified samples.

**Figure 2:**
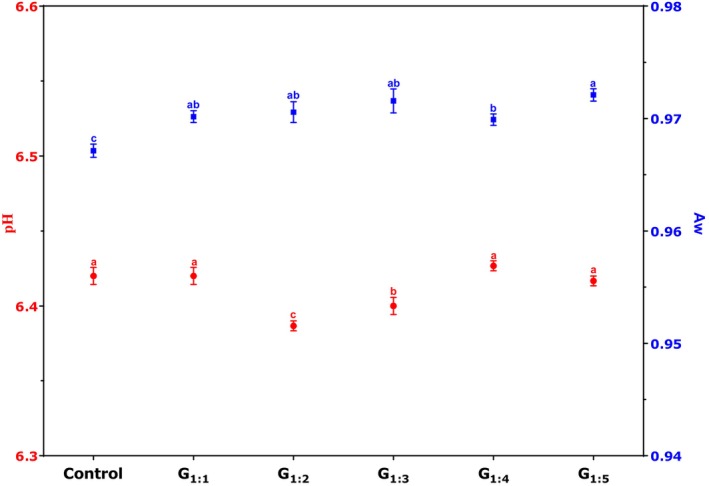
The pH and water activity (Aw) of pâtés with partial replacement of pork fat by hydrated pea protein. Different letters indicate significant differences based on Tukey's test (*P* < 0.05). Error bars depict the standard error of the average. Batches: described in Fig. 1.

Water activity (Aw) and pH values of the pâtés are shown in Fig. 2. The Aw values ranged from 0.96 in the Control to 0.97 in the modified treatments, while pH values remained around 6.4, both typical for this type of meat product.^11^, ^21^ While statistically significant differences were observed among treatments for both parameters (*P* < 0.05), the variations were minor and did not translate into meaningful practical effects. It is worth noting that considerable increases in these parameters could promote microbial growth, accelerating spoilage and compromising microbiological safety. Thus, the reformulations are appropriate, as they maintained these critical safety and quality parameters virtually unchanged.

#### Color and penetration test

**Figure 3:**
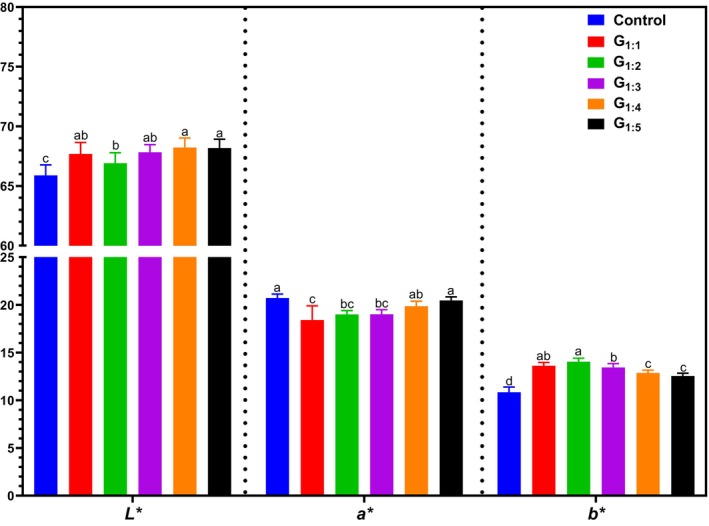
Instrumental color parameters (L*, a*, b*) of canned pâtés formulated with hydrated pea protein as a fat replacer. Different letters indicate significant differences based on Tukey's test (*P* < 0.05). Error bars depict the standard error of the average. Batches: described in Fig. 1.

Figure 3 presents the instrumental color results (L*, a*, and b*) of the pâtés formulated with partial replacement of pork fat by hydrated pea protein concentrate at different protein‐to‐water ratios. Lightness (L*) significantly increased in all reformulated treatments (G_1:1_ to G_1:5_) compared to the Control (*P* < 0.05), particularly in G_1:4_ and G_1:5_, likely due to the higher water content resulting in a lighter appearance. Regarding the a* value (redness), treatments with higher hydration ratios (G_1:4_ and G_1:5_) better preserved the reddish hue than those with lower water content (*P* < 0.05). In contrast, the b* value (yellowness) was highest in G_1:1_ and G_1:2_ (*P* < 0.05), likely due to the inherent yellowish tone of pea protein concentrate. Indeed, this ingredient has a high L* value (91.59 ± 1.98), a strong yellow tendency (b*: 17.79 ± 2.27), and low redness (a*: 1.00 ± 0.52), which explains the observed color changes in reformulated pâtés. Similar effects were reported by Broucke *et al*.^22^ in emulsified sausages and by Colomer Sellas *et al*.^23^ in dry fermented sausages, both showing increased L* and b* and reduced a* values with pea protein inclusion. Likewise, Trindade *et al*.^24^ observed increased lightness and yellowness and slight redness reduction in pâtés with hydrated pea protein, consistent with the present findings. As color directly affects consumer acceptance, such variations must be carefully considered during the development and marketing of reformulated meat products.^25^

**Figure 4:**
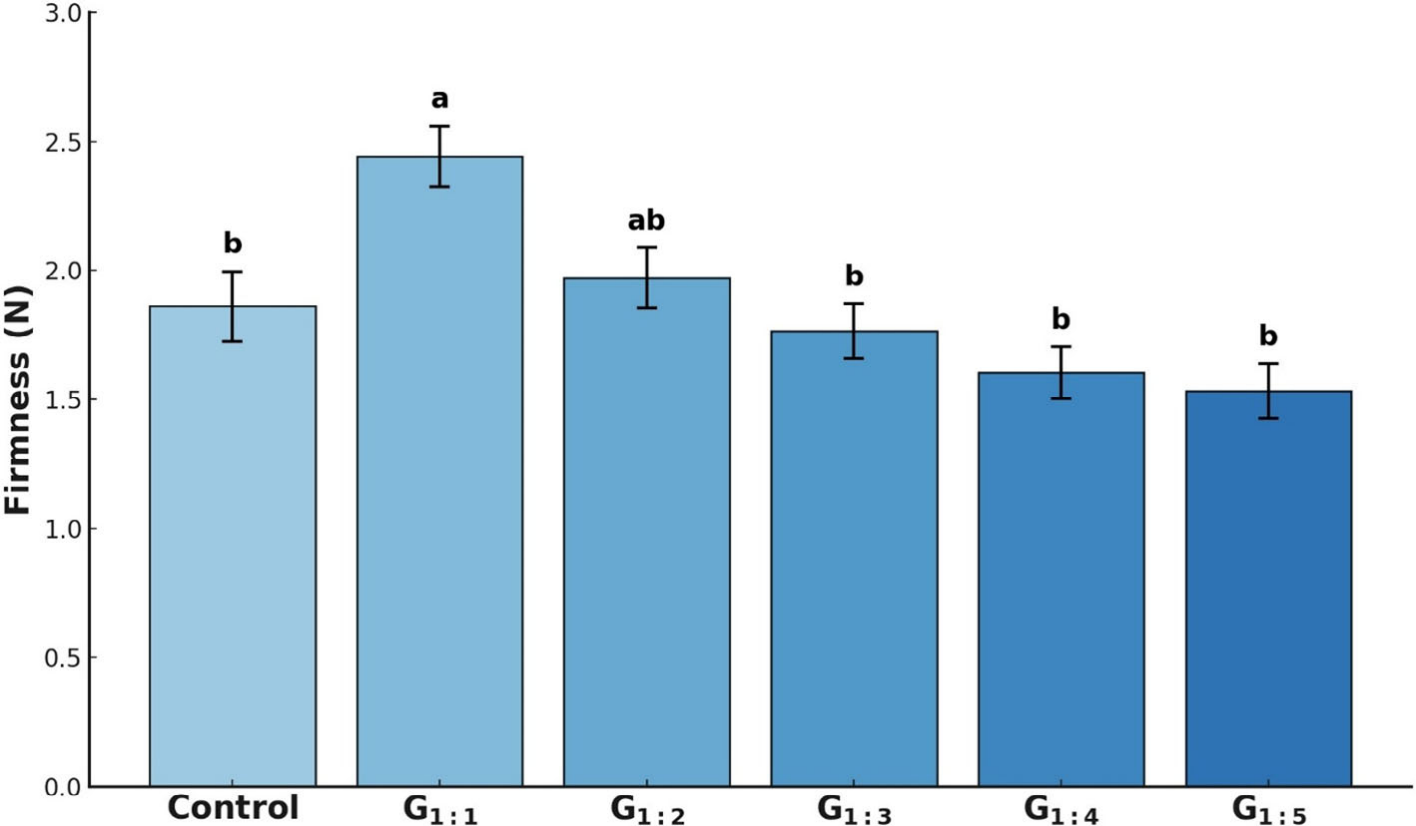
Firmness (N) of canned pâtés as determined by the penetration test. Different letters indicate significant differences based on Tukey's test (*P* < 0.05). Error bars depict the standard error of the average. Batches: described in Fig. 1.

Penetration test results are shown in Fig. 4. Only G_1:1_ exhibited significantly higher firmness than the Control (*P* < 0.05), possibly due to this treatment's higher pea protein content. The higher firmness observed in G_1:1_ can be linked to a denser protein network structure, as explained by Zhang *et al*.,^26^ where protein–protein interactions formed during hydration increased resistance to mechanical deformation. Comparable effects have been observed in other muscle‐based systems. For instance, Yuliarti *et al*.^27^ reported that increasing pea protein levels in meat analogue formulations significantly enhanced hardness, chewiness, and viscoelasticity. At the same time, Zhu *et al*.^28^ found that adding up to 9% pea protein isolate into duck meat batters progressively increased hardness, gumminess, and chewiness. These consistent observations across different systems support the interpretation that higher pea protein content leads to a denser protein matrix and firmer textural quality. However, one of the main challenges in fat reduction is maintaining desirable texture, a key attribute for consumer acceptance.^23^ The fact that G_1:2_ to G_1:5_ exhibited firmness similar to the Control (*P* > 0.05) is promising, suggesting that partial fat replacement with hydrated pea protein can be achieved without compromising this important sensory attribute.

#### Sensory analysis

**Figure 5:**
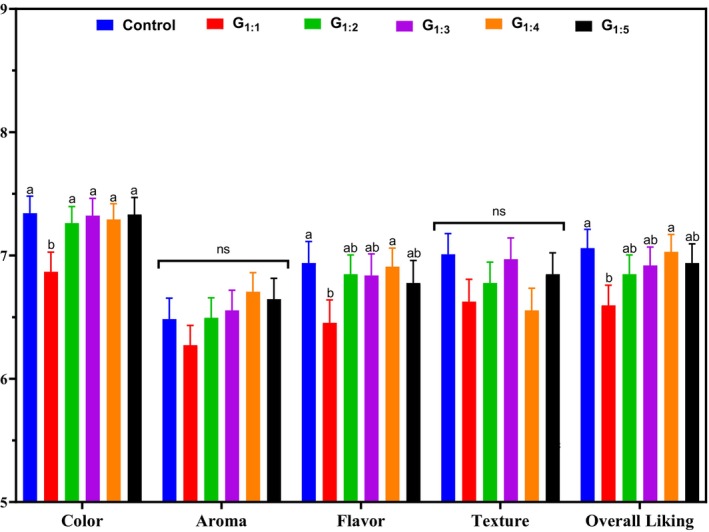
Consumer acceptance scores of pâtés with partial fat replacement. Different letters indicate significant differences based on Tukey's test (*P* < 0.05). Error bars depict the standard error of the average. Batches: described in Fig. 1.

The results of the sensory acceptance test are shown in Fig. 5. No significant differences among treatments were observed in aroma and texture scores (*P* > 0.05). However, G_1:1_ scored significantly lower than the Control in color, flavor, and overall liking (*P* < 0.05). This reduced acceptance may be associated with the higher concentration of pea protein in this formulation, which, when insufficiently hydrated, can intensify vegetal flavors and impair color uniformity and spreadability. Supporting this observation, Shanthakumar *et al*.^11^ noted that legume proteins may carry volatile compounds responsible for grassy, beany, or earthy flavors, which become more pronounced at higher concentrations. In contrast, the other reformulated treatments (G_1:2_ to G_1:5_) received scores similar to the Control for all sensory attributes (*P* > 0.05). These findings suggest that adjusting the hydration level of pea protein is critical to minimizing negative sensory impacts. Future studies could explore the use of natural flavor masking agents, color stabilizers, or pea protein processed to reduce off‐flavors, aiming to improve the sensory quality of formulations with higher protein concentrations.

**Figure 6:**
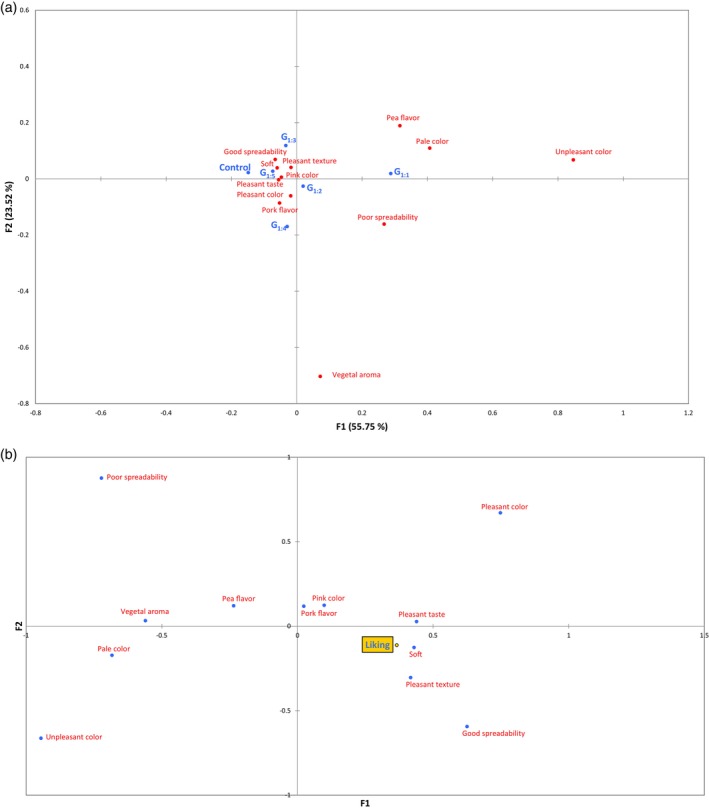
(a) Correspondence analysis of significant CATA descriptors for pâtés. (b) Principal coordinate analysis (PCoA) correlating CATA descriptors with overall liking scores. Batches: described in Fig. 1.

Figure 6(a) shows the correspondence analysis based on CATA data. The first (F1) and second (F2) dimensions explained 55.75% and 23.52% of the total variance, respectively. Samples were grouped along F1 into two main clusters: the Control, G_1:3_, G_1:4_, and G_1:5_ were positioned on the negative side, while G_1:1_ and G_1:2_ were on the positive side, with G_1:2_ located near the center. G_1:1_ was strongly associated with descriptors such as pale color, pea taste, unpleasant color, and poor spreadability, which were negatively correlated with liking (Fig. 6(b)). Conversely, samples located on the negative side of F1 (Control, G_1:3_, G_1:4_, and G_1:5_) were linked to attributes positively associated with liking, such as soft texture, pleasant flavor, and good spreadability (Fig. 6(b)). These results are consistent with instrumental data, particularly texture, demonstrating that treatments with lower levels of pea protein maintained desirable characteristics of the traditional product, supporting better sensory acceptance.

**Figure 7:**
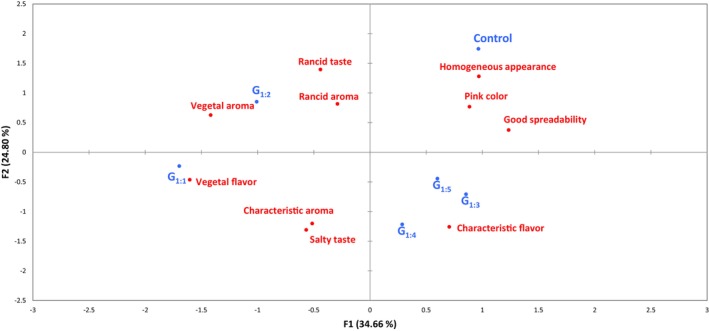
Generalized procrustes analysis (GPA) of sensory profiles of canned pâtés evaluated by trained panelists. Batches: described in Fig. 1.

Figure 7 shows the GPA results from the trained panel sensory profile evaluation. Dimension F1 explained 34.66%, and F2 24.80% of the total variance. The analysis divided samples into two distinct groups along F1. On the positive side were the Control, G_1:3_, G_1:4_, and G_1:5_, associated with positive attributes such as homogeneous appearance, pink color, good spreadability, and characteristic flavor. On the negative side were G_1:1_ and G_1:2_, with G_1:1_ strongly linked to vegetal flavor and G_1:2_ primarily associated with vegetal aroma. These GPA results corroborate the acceptance and CATA findings, emphasizing that balancing pea protein and water proportions is crucial to preserve positive sensory attributes and ensure overall consumer acceptability.

#### Rheological characterization: yield stress and modulus of elasticity

**Figure 8:**
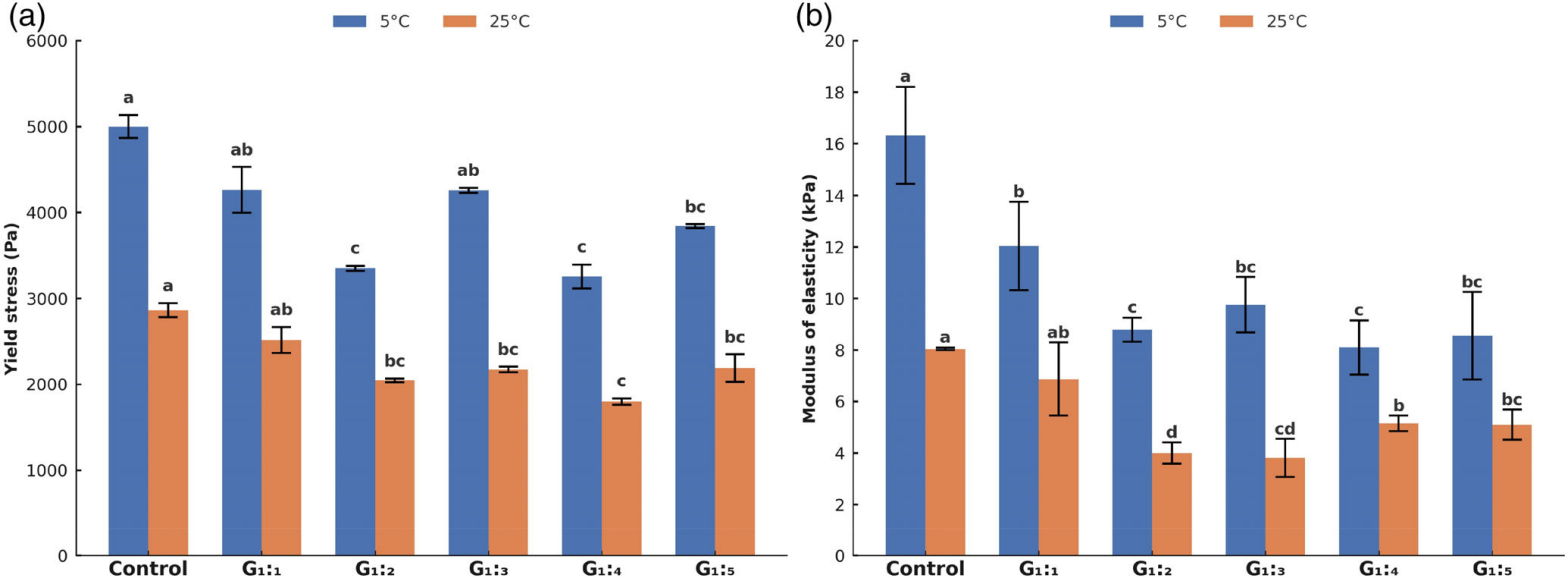
(a) Yield stress (Pa) and (b) modulus of elasticity (kPa) of pâté formulations at 5 °C and 25 °C. Different letters indicate significant differences based on Tukey's test (*P* < 0.05). Error bars depict the standard error of the average. Batches: described in Fig. 1.

The rheological behavior of the pâtés was assessed through yield stress and shear modulus of elasticity at 5 °C and 25 °C (Fig. 8). The yield stress values ranged from 5001 to 3256 Pa at 5 °C and from 2864 to 1797 Pa at 25 °C (Fig. 8(a)). Control and G_1:1_ samples showed the highest yield stress values at both temperatures (*P* < 0.05), indicating a firmer structure with greater resistance to flow initiation. In contrast, G_1:2_, G_1:4_, and G_1:5_ exhibited significantly lower values (*P* < 0.05), suggesting a more fragile structure and lower internal resistance to deformation.^29^, ^30^ G_1:3_ showed an anomalous behavior at 5 °C, with yield stress similar to the Control, but at 25 °C, it followed the same trend of reduced structural resistance observed in the other reformulated samples.

This reduction in yield stress is relevant because it correlates with improved spreadability, a desirable feature in pâtés. Lower yield stress values indicate that the product requires less force to initiate flow, making it easier to spread^29^, ^31^ – a characteristic aligned with the sensory analysis, in which G_1:2_ to G_1:5_ were associated with descriptors such as ‘soft texture’ and ‘good spreadability’. At the same time, G_1:1_ was related to ‘firm texture’ and ‘poor spreadability’. Although G_1:2_ was positioned in a different quadrant in the CATA correspondence analysis, it remained close to the cluster formed by the Control, G_1:3_, G_1:4_, and G_1:5_ samples, suggesting a similar sensory perception. This is consistent with its rheological profile, which also aligned with these samples regarding lower yield stress and modulus of elasticity.

Regarding the shear modulus of elasticity, values ranged from 8.1 to 16.3 kPa at 5 °C and from 3.8 to 8.0 kPa at 25 °C (Fig. 8(b)). As expected, all samples showed lower modulus values at 25 °C, which reflects the softening effect of temperature, particularly due to pork fat transitioning from solid to semi‐liquid state. This thermal transition reduces interparticle friction and weakens the internal network, decreasing structural stiffness.^3^, ^22^, ^24^ Control and G_1:1_ samples retained the highest modulus values (*P* < 0.05), while G_1:2_ to G_1:5_ exhibited significantly lower values (*P* < 0.05), reinforcing the perception of greater spreadability and softer texture in these samples. Similar findings were reported by Chung *et al*.,^29^ who demonstrated that textural softness correlates with lower resistance under applied shear, improving spreadability and consumer liking.

It is important to note that the instrumental penetration test did not detect significant differences in firmness between the Control and most reformulated treatments, except for G_1:1_, which was significantly firmer. This result agrees with the rheological data, confirming that G_1:1_ formed a denser and more cohesive network. However, the similarity in penetration force between G_1:2_ to G_1:5_ and the Control reflects the complex interaction between shear and compression forces, which assess distinct aspects of texture.^3^, ^30^ While the modulus of elasticity captures resistance under shear (significant for spreadability), the penetration test evaluates compression‐related firmness, which may remain less affected by hydration levels.

In summary, the rheological findings support the instrumental and sensory data, indicating that formulations G_1:2_ to G_1:5_ resulted in softer, more spreadable pâtés. The G_1:3_ sample presented an unexpectedly high yield stress at 5 °C, which did not persist at 25 °C, suggesting a possible localized network structuring. Conversely, the G_1:1_ treatment retained greater rigidity and firmness, which was associated with lower sensory acceptance. These results highlight the importance of protein hydration levels in modulating the microstructure and texture of fat‐reduced meat products.

### Analyses performed during storage

#### Microbiological quality

The progression of mesophilic aerobic microorganism counts was significantly (*P* < 0.05) influenced by the interaction between treatment and storage time (Table 1). All treatments showed microbial counts below 1 log CFU g^−1^ immediately after thermal processing, indicating the high effectiveness of the pasteurization process in reducing the initial microbial load. As expected, a gradual increase in microbial counts was observed throughout storage. However, even after 60 days, counts remained low, reaching approximately 4 log CFU g^−1^ in the Control and between 2 and 3 log CFU g^−1^ in the reformulated treatments. These results demonstrate the high microbiological stability of the products, likely due to intrinsic and extrinsic factors, including hermetic sealing, pasteurization, and refrigerated storage – all of which significantly limit microbial growth. Similar findings have been reported by other authors, highlighting the effectiveness of pasteurization combined with refrigeration in maintaining microbial quality in canned meat products stored under similar conditions.^32^

**Table 1:** Counts of mesophilic aerobic microorganisms (log CFU g^−1^) and thiobarbituric acid reactive substances (TBARS) values (mg MDA kg^−1^) of pâtés during 60 days of refrigerated storage

Treatment	Storage day	Mesophilic aerobic microorganism (log CFU g^−1^)	TBARS (mg MDA kg^−1^)
Control	1	< 1.00	0.02^m^
	15	1.0^gh^	0.03^m^
	30	1.24^fgh^	0.09^l^
	45	2.86^abc^	0.08^l^
	60	3.77^a^	0.13^jk^
G_1:1_	1	< 1.00	0.19^fg^
	15	1.39^fgh^	0.25^e^
	30	1.60^efgh^	0.47^b^
	45	1.74^defgh^	0.51^a^
	60	2.48^bcde^	0.53^a^
G_1:2_	1	< 1.00	0.21^fg^
	15	1.0^gh^	0.38^c^
	30	1.60^efgh^	0.40^c^
	45	2.13^cdef^	0.51^ab^
	60	2.15^cdef^	0.53^a^
G_1:3_	1	< 1.00	0.08^l^
	15	1.60^efgh^	0.24^ef^
	30	1.65^defgh^	0.21^efg^
	45	1.95^cdefg^	0.31^d^
	60	3.24^ab^	0.30^d^
G_1:4_	1	< 1.00	0.03^m^
	15	1.45^efgh^	0.16^ij^
	30	1.45^efgh^	0.10^kl^
	45	1.30^fgh^	0.19^gh^
	60	2.7^bcd^	0.20^fg^
G_1:5_	1	< 1.00	0.036 ^m^
	15	0.74^h^	0.189 ^ghi^
	30	1.15^fgh^	0.126 ^jk^
	45	1.59^efgh^	0.157 ^hij^
	60	2.0^cdefg^	0.218 ^efg^
	SEM	0.01	0.005
	T × ST	***	***

Different letters within each column indicate significant differences (*P* < 0.05) according to Tukey's test. Batches: Control: 100% pork fat; Reformulated pâtés: substitution of 50% pork fat with hydrated pea protein using different protein‐to‐water ratios: 1:1 (G_1:1_), 1:2 (G_1:2_), 1:3 (G_1:3_), 1:4 (G_1:4_), and 1:5 (G_1:5_). SEM: standard error of the mean. T × ST: interaction between treatment and storage time; ***P < 0.001.

#### Oxidative stability

TBARS values were significantly influenced (*P* < 0.05) by the interaction between treatment and storage time (Table 1), with reformulated products exhibiting greater lipid oxidation during storage despite having a lower fat content than the Control. TBARS levels increased more sharply as the concentration of pea protein increased across the formulations (*P* < 0.05). Protein–lipid co‐oxidation mechanisms can explain the observed increase in TBARS values with higher concentrations of pea protein. Pea protein is susceptible to oxidative degradation, forming reactive intermediates such as protein radicals and carbonyl compounds that can initiate or propagate lipid peroxidation.^33^, ^34^ In fact, oxidized protein species are known to catalyze lipid oxidation in mixed food matrices, correlating with increased TBARS levels.^33^ Moreover, plant protein isolates often contain trace pro‐oxidant elements, such as iron and copper, which may accelerate lipid oxidation in emulsified systems.^35^ Nevertheless, treatments with higher hydration ratios of pea protein (particularly G_1:4_ and G_1:5_) exhibited TBARS values that, although statistically higher than those of the Control (*P* < 0.05), remained sufficiently low to avoid any negative impact on the product's sensory quality or safety.^36^

## CONCLUSION

This study demonstrated that partially replacing pork fat with hydrated pea protein concentrate at optimal hydration ratios (particularly 1:3, 1:4, and 1:5) effectively reduced lipid content and increased protein levels in canned pâtés without negatively impacting technological performance or sensory quality. Additionally, microbiological quality and oxidative stability were preserved over 60 days of refrigerated storage. Hydration level was identified as a critical factor for balancing product texture and consumer acceptance. From a practical standpoint, these findings offer the meat industry a viable strategy for developing healthier pâtés aligned with consumer preferences for nutritious foods. Future studies should investigate alternative hydration approaches, such as enzymatic treatments or fermentation, aiming to neutralize or mask the vegetal off‐flavors and enhance the protein's functionality. Additionally, combining pea protein with ingredients known to improve flavor, such as natural umami enhancers, spices, herbs, or antioxidant‐rich extracts, may further improve the sensory acceptability and overall quality of reformulated products.

## Supporting information


**Table S1.** Definitions and reference standards for descriptive sensory terms used in canned pâtés.

## Data Availability

The data that support the findings of this study are available from the corresponding author upon reasonable request.
