# Supplementary material for: Physicochemical, rheological, sensory, microbiological, and oxidative properties of canned pâtés reformulated with hydrated pea protein as a fat replacer
Source: J Sci Food Agric. 2025 Nov 17;106(4):2231–41. doi: 10.1002/jsfa.70331 (PMC12872238; doi:10.1002/jsfa.70331)
Supplement: Supplementary file 1 — Table S1. Definitions and reference standards for descriptive sensory terms used in canned pâtés. [file JSFA-106-2231-s001.docx]

| Supplementary Table S1 – Definitions and reference standards for descriptive sensory terms used in canned pâtés |
| --- |
| APPEARANCE |
| Homogeneous appearance: Smooth appearance, without visible roughness on the pâté surface. |
| Low: Canned liver pâté (Molto) |
| High: Squeezable ham pâté (Excelsior) |
| Pink color: Characteristic pink color of pork meat pâté. |
| Low: Pâté made with pork meat and no added coloring. |
| High: Pâté made with pork meat and 0.8% added coloring. |
| Spreadability: Ease with which the pâté can be spread. |
| Low: Unsalted extra butter (Santa Clara) |
| High: Cream cheese (Elegê) |
| AROMA |
| Vegetal aroma: Characteristic aroma of green legumes. |
| None: Water at room temperature |
| High: Pea protein (hydration ratio 1:5) |
| Characteristic aroma: Characteristic aroma of pork meat pâté. |
| Low: 1 g of Excelsior ham pâté with 10 mL water added. |
| High: Squeezable ham pâté (Excelsior) |
| Rancid aroma: Intensity of odor characteristic of oxidized pork fat. |
| None: Water at room temperature |
| High: Pork fat stored for 6 months |
| FLAVOR |
| Salty taste: Intensity of salty taste perceived when tasting a portion of pâté. |
| None: Squeezable ham pâté (Excelsior) |
| High: 10 g of Excelsior ham pâté with 0.5% added salt |
| Rancid taste: Intensity of flavor characteristic of oxidized pork fat. |
| None: Water at room temperature |
| High: Pork fat stored for 6 months |
| Characteristic taste: Intensity of flavor typical of pork meat pâté. |
| Low: Canned meat pâté (La Negra) |
| High: Squeezable ham pâté (Excelsior) |
| Vegetal taste: Intensity of vegetal residual taste (reminiscent of green legumes) perceived when tasting a portion of pâté. |
| Low: Squeezable ham pâté (Excelsior) |
| High: Pea protein (hydration ratio 1:5) |
